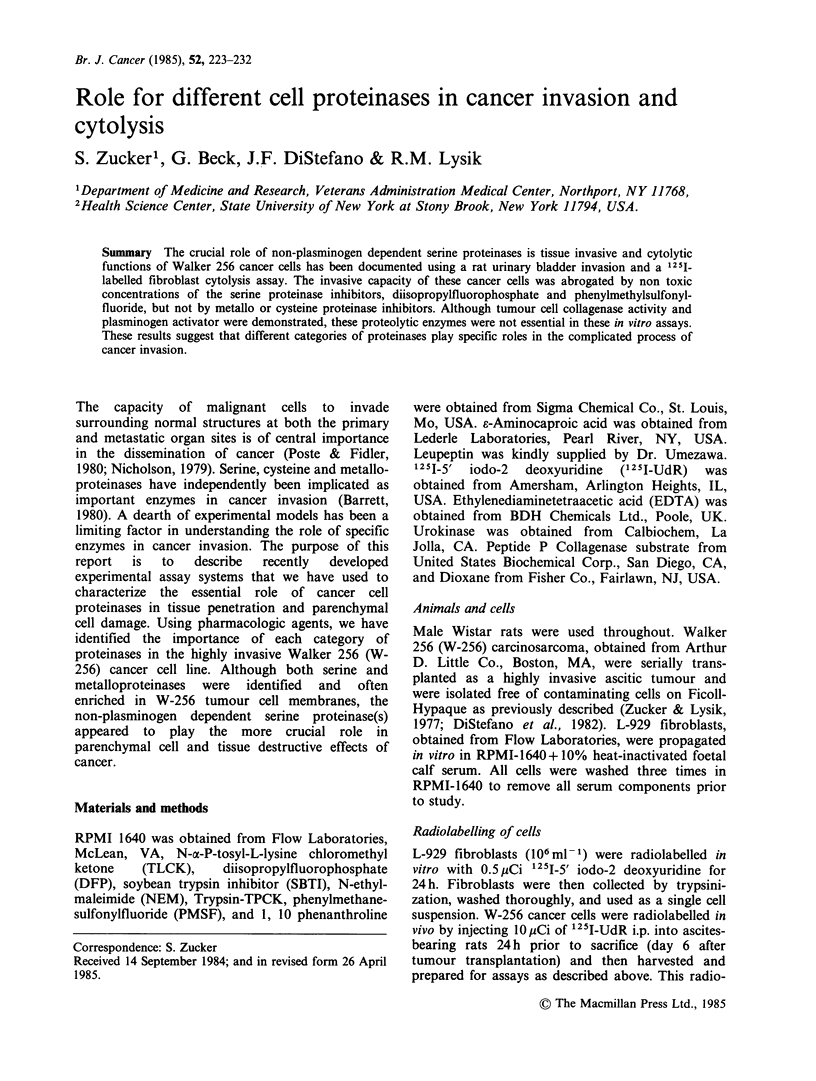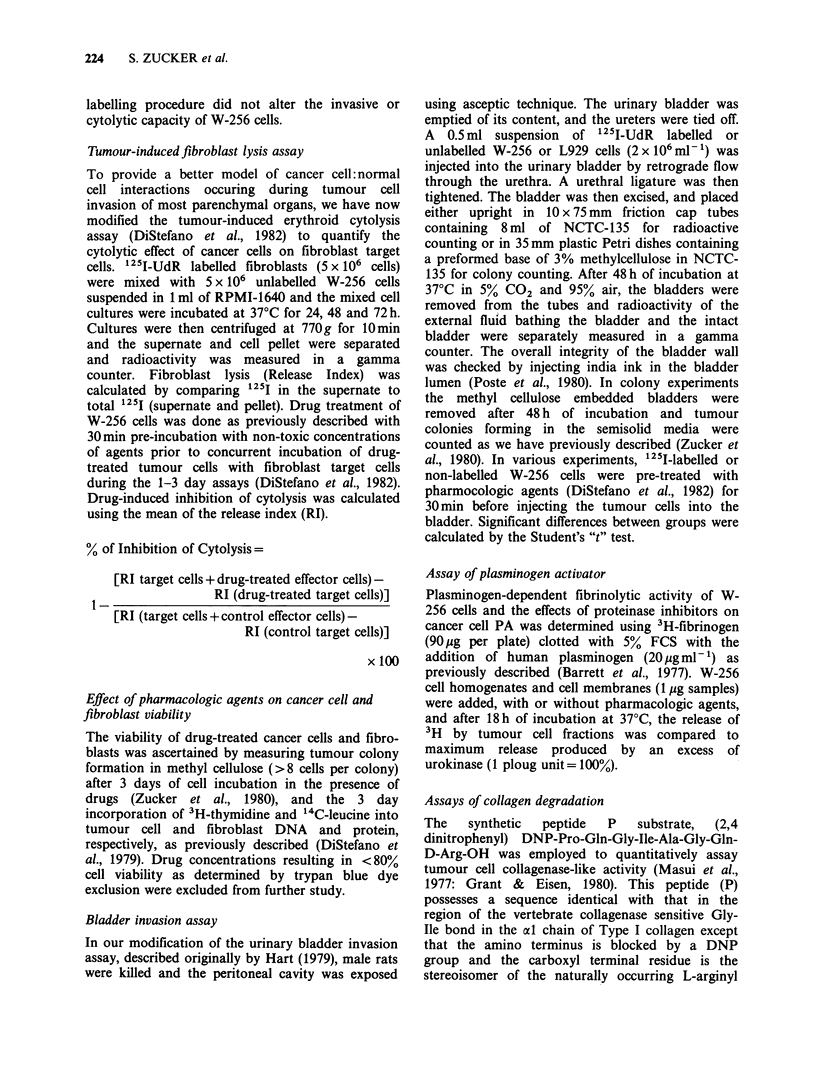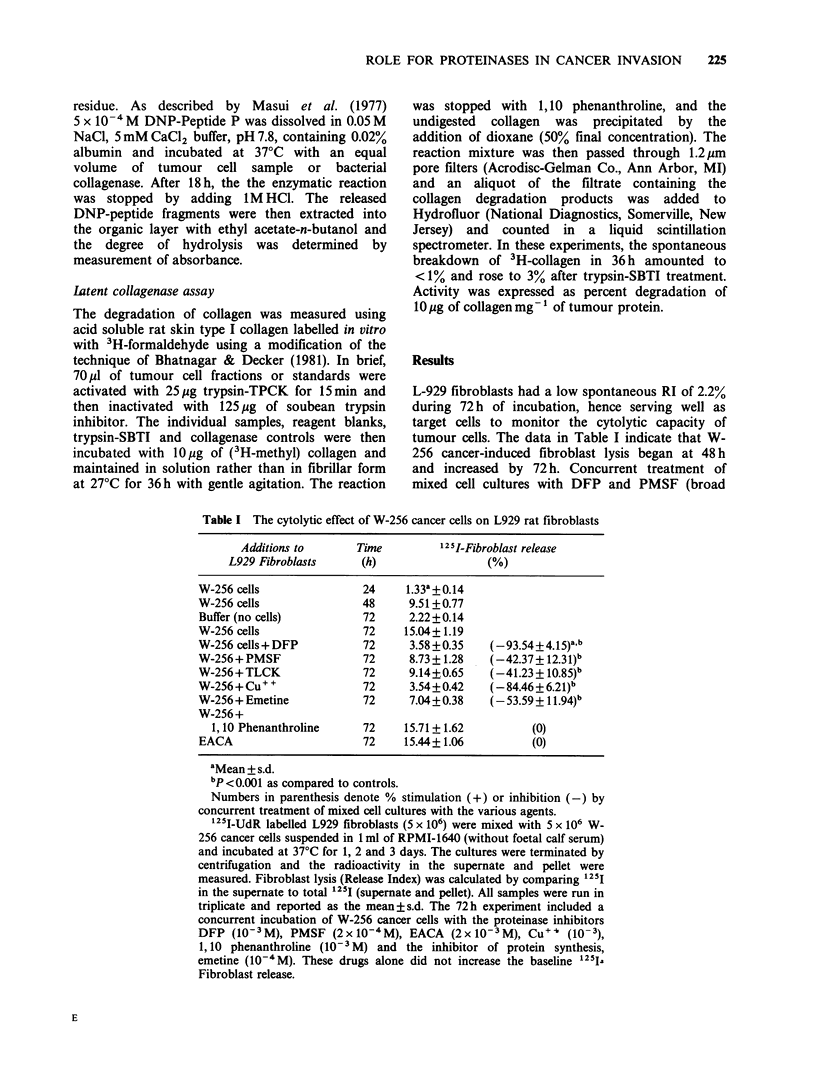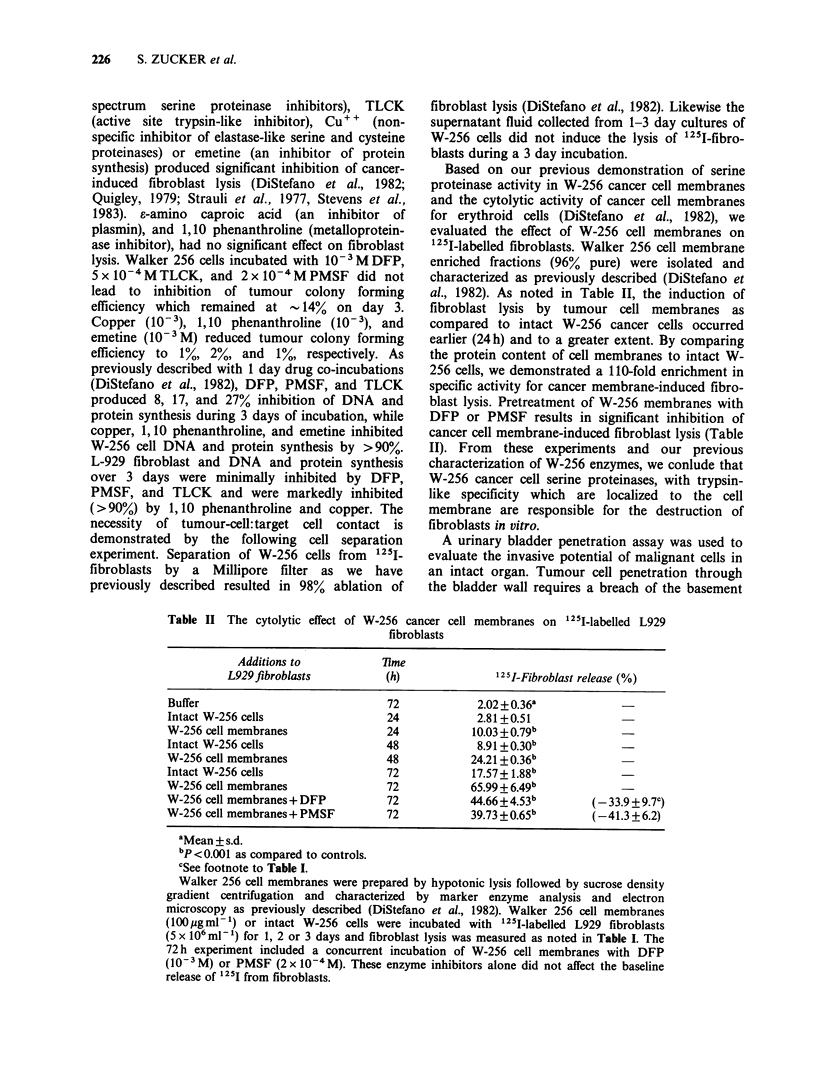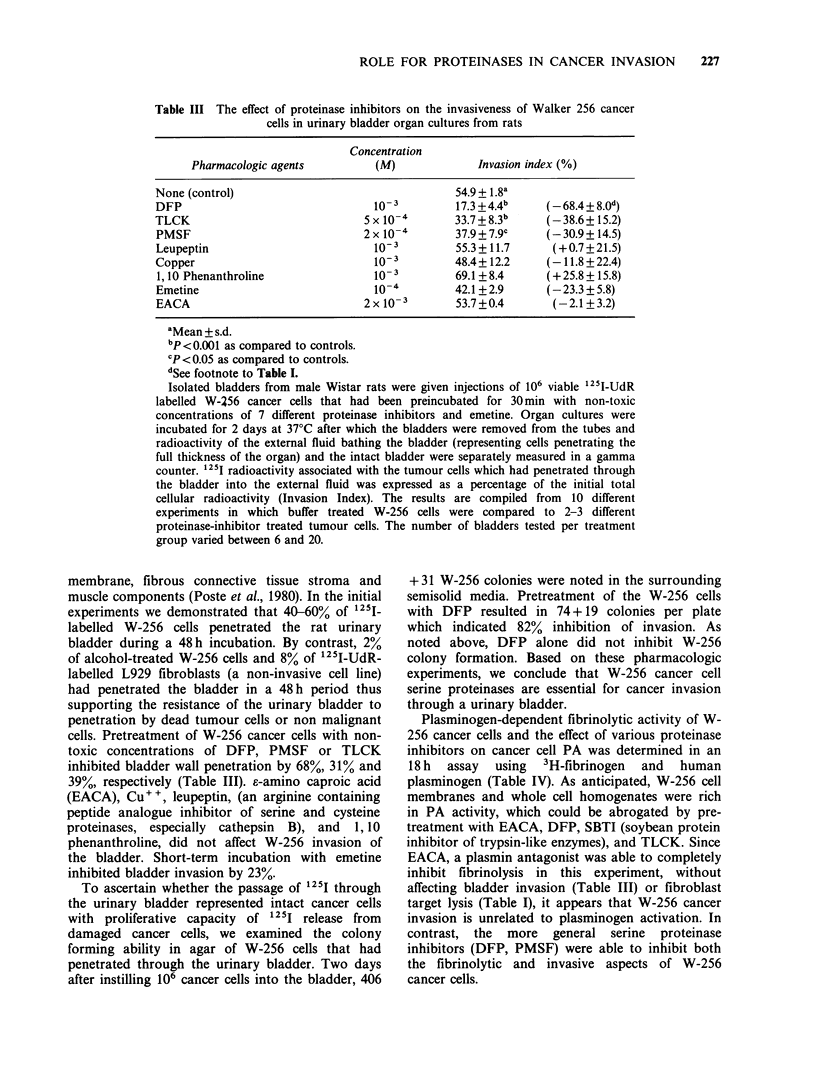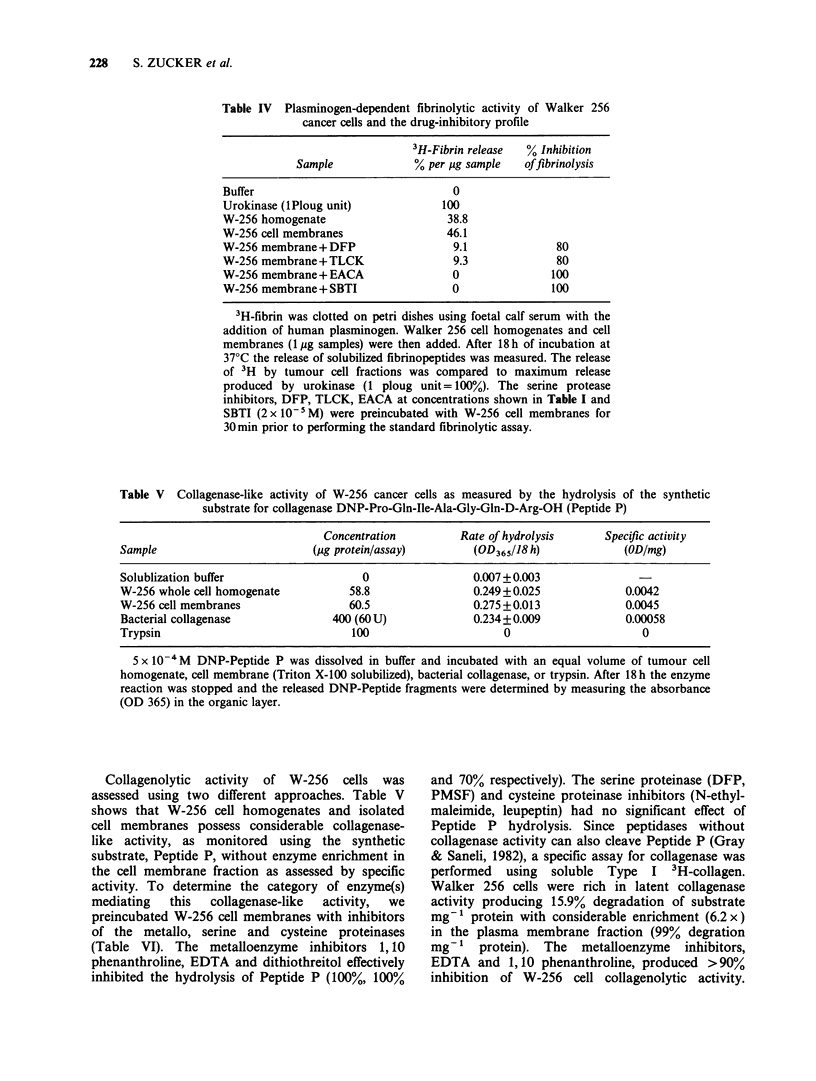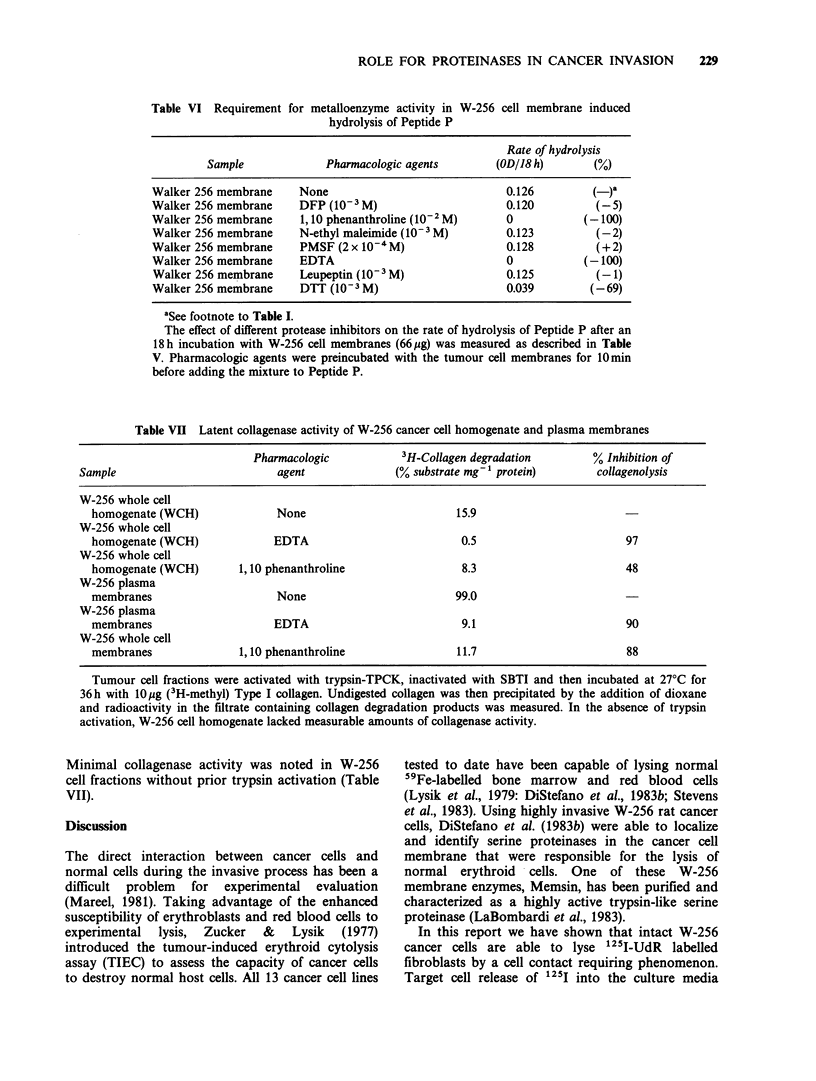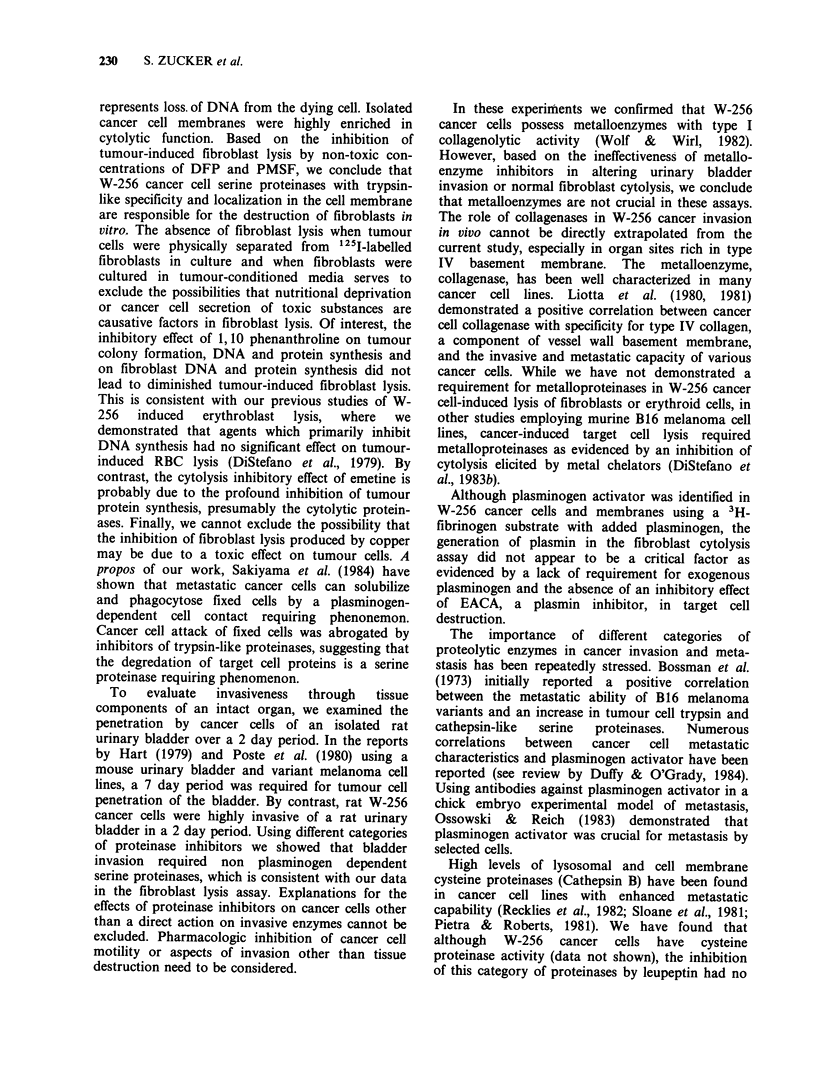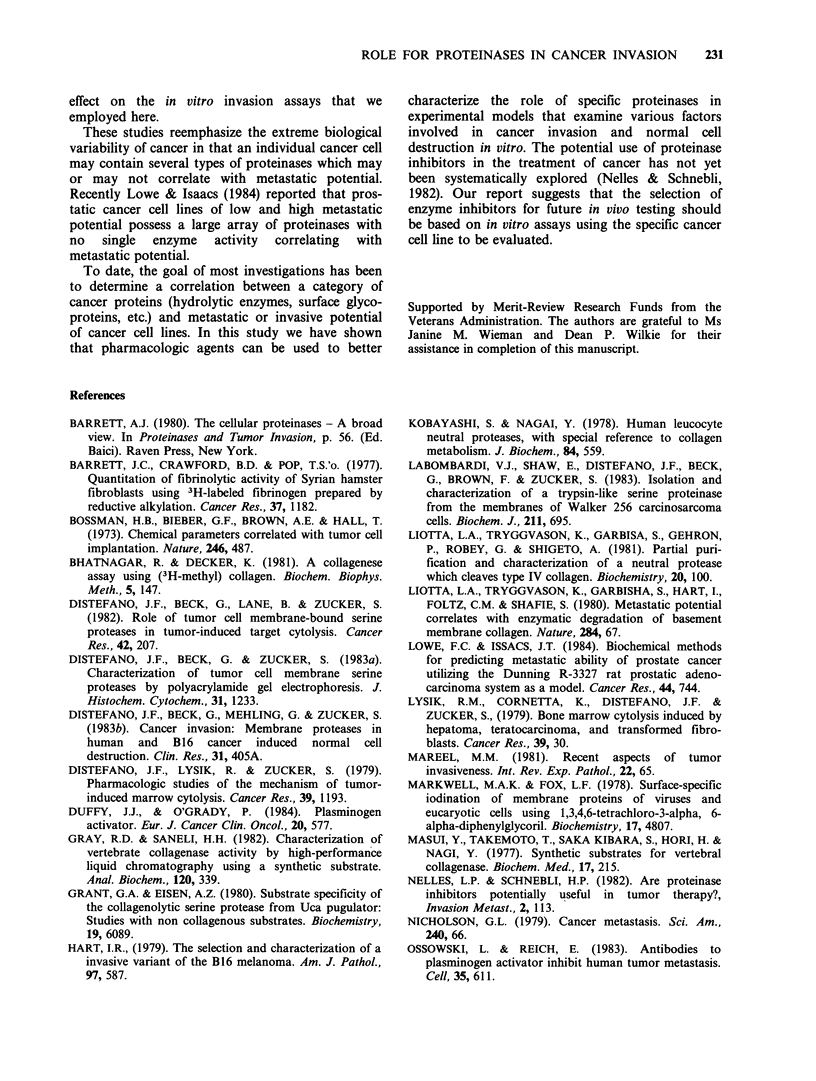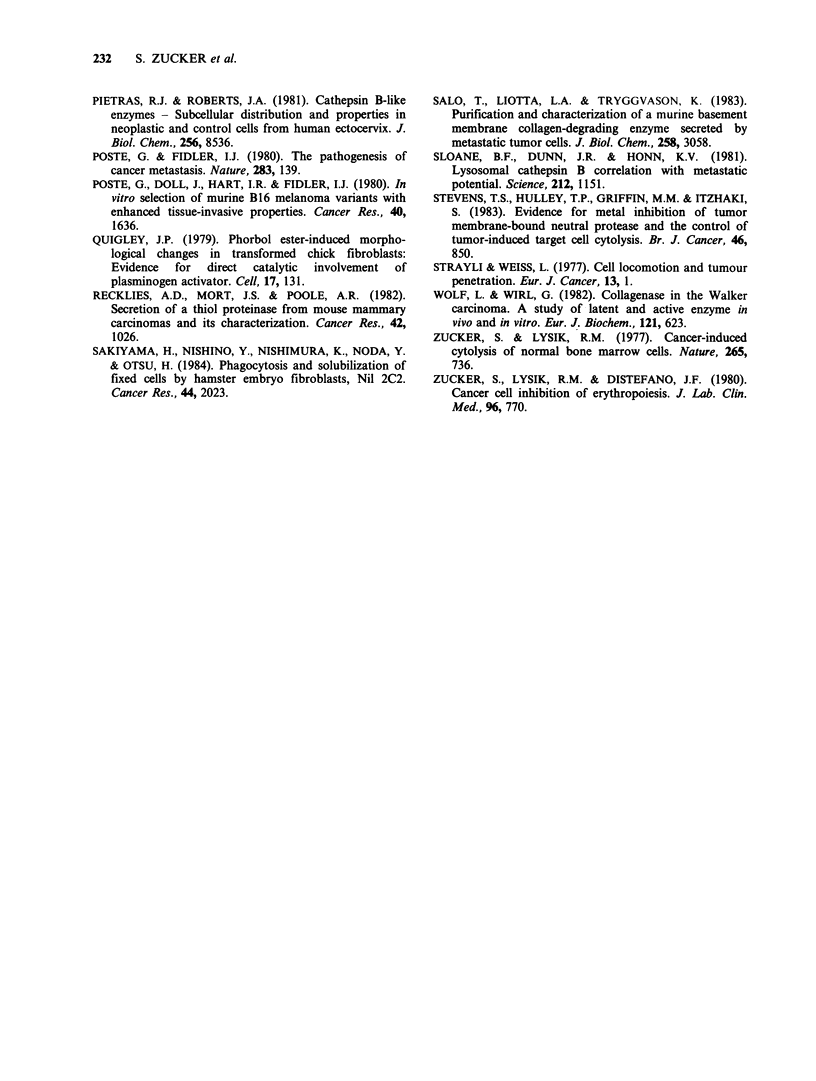# Role for different cell proteinases in cancer invasion and cytolysis.

**DOI:** 10.1038/bjc.1985.181

**Published:** 1985-08

**Authors:** S. Zucker, G. Beck, J. F. DiStefano, R. M. Lysik

## Abstract

The crucial role of non-plasminogen dependent serine proteinases is tissue invasive and cytolytic functions of Walker 256 cancer cells has been documented using a rat urinary bladder invasion and a 125I-labelled fibroblast cytolysis assay. The invasive capacity of these cancer cells was abrogated by non toxic concentrations of the serine proteinase inhibitors, diisopropylfluorophosphate and phenylmethylsulfonylfluoride, but not by metallo or cysteine proteinase inhibitors. Although tumour cell collagenase activity and plasminogen activator were demonstrated, these proteolytic enzymes were not essential in these in vitro assays. These results suggest that different categories of proteinases play specific roles in the complicated process of cancer invasion.


*Br. J. Cancer* (1985), **52**, 223–232

### Role for different cell proteinases in cancer invasion and cytolysis

S. Zucker[1], G. Beck, J.F. DiStefano & R.M. Lysik

[1]*Department of Medicine and Research, Veterans Administration Medical Center, Northport, NY 11768,*
[2]*Health Science Center, State University of New York at Stony Brook, New York 11794, USA.*

**Summary** The crucial role of non-plasminogen dependent serine proteinases is tissue invasive and cytolytic functions of Walker 256 cancer cells has been documented using a rat urinary bladder invasion and a $^{125}$I-labelled fibroblast cytolysis assay. The invasive capacity of these cancer cells was abrogated by non toxic concentrations of the serine proteinase inhibitors, diisopropylfluorophosphate and phenylmethylsulfonylfluoride, but not by metallo or cysteine proteinase inhibitors. Although tumour cell collagenase activity and plasminogen activator were demonstrated, these proteolytic enzymes were not essential in these *in vitro* assays. These results suggest that different categories of proteinases play specific roles in the complicated process of cancer invasion.

The capacity of malignant cells to invade surrounding normal structures at both the primary and metastatic organ sites is of central importance in the dissemination of cancer (Poste & Fidler, 1980; Nicholson, 1979). Serine, cysteine and metalloproteinases have independently been implicated as important enzymes in cancer invasion (Barrett, 1980). A dearth of experimental models has been a limiting factor in understanding the role of specific enzymes in cancer invasion. The purpose of this report is to describe recently developed experimental assay systems that we have used to characterize the essential role of cancer cell proteinases in tissue penetration and parenchymal cell damage. Using pharmacologic agents, we have identified the importance of each category of proteinases in the highly invasive Walker 256 (W-256) cancer cell line. Although both serine and metalloproteinases were identified and often enriched in W-256 tumour cell membranes, the non-plasminogen dependent serine proteinase(s) appeared to play the more crucial role in parenchymal cell and tissue destructive effects of cancer.

### Materials and methods

RPMI 1640 was obtained from Flow Laboratories, McLean, VA, N-α-P-tosyl-L-lysine chloromethyl ketone (TLCK), diisopropylfluorophosphate (DFP), soybean trypsin inhibitor (SBTI), N-ethylmaleimide (NEM), Trypsin-TPCK, phenylmethanesulfonylfluoride (PMSF), and 1, 10 phenanthroline were obtained from Sigma Chemical Co., St. Louis, Mo, USA. ε-Aminocaproic acid was obtained from Lederle Laboratories, Pearl River, NY, USA. Leupeptin was kindly supplied by Dr. Umezawa. $^{125}$I-5′ iodo-2 deoxyuridine ($^{125}$I-UdR) was obtained from Amersham, Arlington Heights, IL, USA. Ethylenediaminetetraacetic acid (EDTA) was obtained from BDH Chemicals Ltd., Poole, UK. Urokinase was obtained from Calbiochem, La Jolla, CA. Peptide P Collagenase substrate from United States Biochemical Corp., San Diego, CA, and Dioxane from Fisher Co., Fairlawn, NJ, USA.

*Animals and cells*

Male Wistar rats were used throughout. Walker 256 (W-256) carcinosarcoma, obtained from Arthur D. Little Co., Boston, MA, were serially transplanted as a highly invasive ascitic tumour and were isolated free of contaminating cells on Ficoll-Hypaque as previously described (Zucker & Lysik, 1977; DiStefano *et al.*, 1982). L-929 fibroblasts, obtained from Flow Laboratories, were propagated *in vitro* in RPMI-1640 + 10% heat-inactivated foetal calf serum. All cells were washed three times in RPMI-1640 to remove all serum components prior to study.

*Radiolabelling of cells*

L-929 fibroblasts ($10^6\,ml^{-1}$) were radiolabelled *in vitro* with 0.5 $\mu$Ci $^{125}$I-5′ iodo-2 deoxyuridine for 24 h. Fibroblasts were then collected by trypsinization, washed thoroughly, and used as a single cell suspension. W-256 cancer cells were radiolabelled *in vivo* by injecting 10 $\mu$Ci of $^{125}$I-UdR i.p. into ascites-bearing rats 24 h prior to sacrifice (day 6 after tumour transplantation) and then harvested and prepared for assays as described above. This radio-

Correspondence: S. Zucker
Received 14 September 1984; and in revised form 26 April 1985.

© The Macmillan Press Ltd., 1985

labelling procedure did not alter the invasive or cytolytic capacity of W-256 cells.

*Tumour-induced fibroblast lysis assay*

To provide a better model of cancer cell:normal cell interactions occuring during tumour cell invasion of most parenchymal organs, we have now modified the tumour-induced erythroid cytolysis assay (DiStefano *et al.*, 1982) to quantify the cytolytic effect of cancer cells on fibroblast target cells. $^{125}$I-UdR labelled fibroblasts ($5 \times 10^6$ cells) were mixed with $5 \times 10^6$ unlabelled W-256 cells suspended in 1 ml of RPMI-1640 and the mixed cell cultures were incubated at 37°C for 24, 48 and 72 h. Cultures were then centrifuged at 770 *g* for 10 min and the supernate and cell pellet were separated and radioactivity was measured in a gamma counter. Fibroblast lysis (Release Index) was calculated by comparing $^{125}$I in the supernate to total $^{125}$I (supernate and pellet). Drug treatment of W-256 cells was done as previously described with 30 min pre-incubation with non-toxic concentrations of agents prior to concurrent incubation of drug-treated tumour cells with fibroblast target cells during the 1–3 day assays (DiStefano *et al.*, 1982). Drug-induced inhibition of cytolysis was calculated using the mean of the release index (RI).

% of Inhibition of Cytolysis =

$$1 - \frac{[\text{RI target cells} + \text{drug-treated effector cells}) - \text{RI (drug-treated target cells)}]}{[\text{RI (target cells} + \text{control effector cells}) - \text{RI (control target cells)}]} \times 100$$

*Effect of pharmacologic agents on cancer cell and fibroblast viability*

The viability of drug-treated cancer cells and fibroblasts was ascertained by measuring tumour colony formation in methyl cellulose (>8 cells per colony) after 3 days of cell incubation in the presence of drugs (Zucker *et al.*, 1980), and the 3 day incorporation of $^{3}$H-thymidine and $^{14}$C-leucine into tumour cell and fibroblast DNA and protein, respectively, as previously described (DiStefano *et al.*, 1979). Drug concentrations resulting in <80% cell viability as determined by trypan blue dye exclusion were excluded from further study.

*Bladder invasion assay*

In our modification of the urinary bladder invasion assay, described originally by Hart (1979), male rats were killed and the peritoneal cavity was exposed using asceptic technique. The urinary bladder was emptied of its content, and the ureters were tied off. A 0.5 ml suspension of $^{125}$I-UdR labelled or unlabelled W-256 or L929 cells ($2 \times 10^6\,\text{ml}^{-1}$) was injected into the urinary bladder by retrograde flow through the urethra. A urethral ligature was then tightened. The bladder was then excised, and placed either upright in 10 × 75 mm friction cap tubes containing 8 ml of NCTC-135 for radioactive counting or in 35 mm plastic Petri dishes containing a preformed base of 3% methylcellulose in NCTC-135 for colony counting. After 48 h of incubation at 37°C in 5% $CO_2$ and 95% air, the bladders were removed from the tubes and radioactivity of the external fluid bathing the bladder and the intact bladder were separately measured in a gamma counter. The overall integrity of the bladder wall was checked by injecting india ink in the bladder lumen (Poste *et al.*, 1980). In colony experiments the methyl cellulose embedded bladders were removed after 48 h of incubation and tumour colonies forming in the semisolid media were counted as we have previously described (Zucker *et al.*, 1980). In various experiments, $^{125}$I-labelled or non-labelled W-256 cells were pre-treated with pharmocologic agents (DiStefano *et al.*, 1982) for 30 min before injecting the tumour cells into the bladder. Significant differences between groups were calculated by the Student's "*t*" test.

*Assay of plasminogen activator*

Plasminogen-dependent fibrinolytic activity of W-256 cells and the effects of proteinase inhibitors on cancer cell PA was determined using $^{3}$H-fibrinogen (90 $\mu$g per plate) clotted with 5% FCS with the addition of human plasminogen (20 $\mu\text{g ml}^{-1}$) as previously described (Barrett *et al.*, 1977). W-256 cell homogenates and cell membranes (1 $\mu$g samples) were added, with or without pharmacologic agents, and after 18 h of incubation at 37°C, the release of $^{3}$H by tumour cell fractions was compared to maximum release produced by an excess of urokinase (1 ploug unit = 100%).

*Assays of collagen degradation*

The synthetic peptide P substrate, (2,4 dinitrophenyl) DNP-Pro-Gln-Gly-Ile-Ala-Gly-Gln-D-Arg-OH was employed to quantitatively assay tumour cell collagenase-like activity (Masui *et al.*, 1977: Grant & Eisen, 1980). This peptide (P) possesses a sequence identical with that in the region of the vertebrate collagenase sensitive Gly-Ile bond in the $\alpha$1 chain of Type I collagen except that the amino terminus is blocked by a DNP group and the carboxyl terminal residue is the stereoisomer of the naturally occurring L-arginyl residue. As described by Masui *et al.* (1977) $5 \times 10^{-4}$ M DNP-Peptide P was dissolved in 0.05 M NaCl, 5 mM $CaCl_2$ buffer, pH 7.8, containing 0.02% albumin and incubated at 37°C with an equal volume of tumour cell sample or bacterial collagenase. After 18 h, the the enzymatic reaction was stopped by adding 1M HCl. The released DNP-peptide fragments were then extracted into the organic layer with ethyl acetate-*n*-butanol and the degree of hydrolysis was determined by measurement of absorbance.

*Latent collagenase assay*

The degradation of collagen was measured using acid soluble rat skin type I collagen labelled *in vitro* with $^3$H-formaldehyde using a modification of the technique of Bhatnagar & Decker (1981). In brief, 70 μl of tumour cell fractions or standards were activated with 25 μg trypsin-TPCK for 15 min and then inactivated with 125 μg of soubean trypsin inhibitor. The individual samples, reagent blanks, trypsin-SBTI and collagenase controls were then incubated with 10 μg of ($^3$H-methyl) collagen and maintained in solution rather than in fibrillar form at 27°C for 36 h with gentle agitation. The reaction was stopped with 1,10 phenanthroline, and the undigested collagen was precipitated by the addition of dioxane (50% final concentration). The reaction mixture was then passed through 1.2 μm pore filters (Acrodisc-Gelman Co., Ann Arbor, MI) and an aliquot of the filtrate containing the collagen degradation products was added to Hydrofluor (National Diagnostics, Somerville, New Jersey) and counted in a liquid scintillation spectrometer. In these experiments, the spontaneous breakdown of $^3$H-collagen in 36 h amounted to <1% and rose to 3% after trypsin-SBTI treatment. Activity was expressed as percent degradation of 10 μg of collagen $mg^{-1}$ of tumour protein.

### Results

L-929 fibroblasts had a low spontaneous RI of 2.2% during 72 h of incubation, hence serving well as target cells to monitor the cytolytic capacity of tumour cells. The data in Table I indicate that W-256 cancer-induced fibroblast lysis began at 48 h and increased by 72 h. Concurrent treatment of mixed cell cultures with DFP and PMSF (broad

**Table I** The cytolytic effect of W-256 cancer cells on L929 rat fibroblasts

| *Additions to L929 Fibroblasts* | *Time (h)* | *$^{125}$I-Fibroblast release (%)* | |
|---|---|---|---|
| W-256 cells | 24 | 1.33[a] ± 0.14 | |
| W-256 cells | 48 | 9.51 ± 0.77 | |
| Buffer (no cells) | 72 | 2.22 ± 0.14 | |
| W-256 cells | 72 | 15.04 ± 1.19 | |
| W-256 cells + DFP | 72 | 3.58 ± 0.35 | (−93.54 ± 4.15)[a,b] |
| W-256 + PMSF | 72 | 8.73 ± 1.28 | (−42.37 ± 12.31)[b] |
| W-256 + TLCK | 72 | 9.14 ± 0.65 | (−41.23 ± 10.85)[b] |
| W-256 + $Cu^{++}$ | 72 | 3.54 ± 0.42 | (−84.46 ± 6.21)[b] |
| W-256 + Emetine | 72 | 7.04 ± 0.38 | (−53.59 ± 11.94)[b] |
| W-256 + 1, 10 Phenanthroline | 72 | 15.71 ± 1.62 | (0) |
| EACA | 72 | 15.44 ± 1.06 | (0) |

[a]Mean ± s.d.
[b]$P < 0.001$ as compared to controls.
Numbers in parenthesis denote % stimulation (+) or inhibition (−) by concurrent treatment of mixed cell cultures with the various agents.
$^{125}$I-UdR labelled L929 fibroblasts ($5 \times 10^6$) were mixed with $5 \times 10^6$ W-256 cancer cells suspended in 1 ml of RPMI-1640 (without foetal calf serum) and incubated at 37°C for 1, 2 and 3 days. The cultures were terminated by centrifugation and the radioactivity in the supernate and pellet were measured. Fibroblast lysis (Release Index) was calculated by comparing $^{125}$I in the supernate to total $^{125}$I (supernate and pellet). All samples were run in triplicate and reported as the mean ± s.d. The 72 h experiment included a concurrent incubation of W-256 cancer cells with the proteinase inhibitors DFP ($10^{-3}$ M), PMSF ($2 \times 10^{-4}$ M), EACA ($2 \times 10^{-3}$ M), $Cu^{++}$ ($10^{-3}$), 1, 10 phenanthroline ($10^{-3}$ M) and the inhibitor of protein synthesis, emetine ($10^{-4}$ M). These drugs alone did not increase the baseline $^{125}$I-Fibroblast release.

spectrum serine proteinase inhibitors), TLCK (active site trypsin-like inhibitor), $Cu^{++}$ (non-specific inhibitor of elastase-like serine and cysteine proteinases) or emetine (an inhibitor of protein synthesis) produced significant inhibition of cancer-induced fibroblast lysis (DiStefano *et al.*, 1982; Quigley, 1979; Strauli *et al.*, 1977, Stevens *et al.*, 1983). ε-amino caproic acid (an inhibitor of plasmin), and 1,10 phenanthroline (metalloproteinase inhibitor), had no significant effect on fibroblast lysis. Walker 256 cells incubated with $10^{-3}$ M DFP, $5 \times 10^{-4}$ M TLCK, and $2 \times 10^{-4}$ M PMSF did not lead to inhibition of tumour colony forming efficiency which remained at ~14% on day 3. Copper ($10^{-3}$), 1,10 phenanthroline ($10^{-3}$), and emetine ($10^{-3}$ M) reduced tumour colony forming efficiency to 1%, 2%, and 1%, respectively. As previously described with 1 day drug co-incubations (DiStefano *et al.*, 1982), DFP, PMSF, and TLCK produced 8, 17, and 27% inhibition of DNA and protein synthesis during 3 days of incubation, while copper, 1,10 phenanthroline, and emetine inhibited W-256 cell DNA and protein synthesis by >90%. L-929 fibroblast and DNA and protein synthesis over 3 days were minimally inhibited by DFP, PMSF, and TLCK and were markedly inhibited (>90%) by 1,10 phenanthroline and copper. The necessity of tumour-cell:target cell contact is demonstrated by the following cell separation experiment. Separation of W-256 cells from $^{125}$I-fibroblasts by a Millipore filter as we have previously described resulted in 98% ablation of fibroblast lysis (DiStefano *et al.*, 1982). Likewise the supernatant fluid collected from 1–3 day cultures of W-256 cells did not induce the lysis of $^{125}$I-fibroblasts during a 3 day incubation.

Based on our previous demonstration of serine proteinase activity in W-256 cancer cell membranes and the cytolytic activity of cancer cell membranes for erythroid cells (DiStefano *et al.*, 1982), we evaluated the effect of W-256 cell membranes on $^{125}$I-labelled fibroblasts. Walker 256 cell membrane enriched fractions (96% pure) were isolated and characterized as previously described (DiStefano *et al.*, 1982). As noted in Table II, the induction of fibroblast lysis by tumour cell membranes as compared to intact W-256 cancer cells occurred earlier (24 h) and to a greater extent. By comparing the protein content of cell membranes to intact W-256 cells, we demonstrated a 110-fold enrichment in specific activity for cancer membrane-induced fibroblast lysis. Pretreatment of W-256 membranes with DFP or PMSF results in significant inhibition of cancer cell membrane-induced fibroblast lysis (Table II). From these experiments and our previous characterization of W-256 enzymes, we conlude that W-256 cancer cell serine proteinases, with trypsin-like specificity which are localized to the cell membrane are responsible for the destruction of fibroblasts *in vitro*.

A urinary bladder penetration assay was used to evaluate the invasive potential of malignant cells in an intact organ. Tumour cell penetration through the bladder wall requires a breach of the basement

**Table II** The cytolytic effect of W-256 cancer cell membranes on $^{125}$I-labelled L929 fibroblasts

| *Additions to L929 fibroblasts* | *Time (h)* | *$^{125}$I-Fibroblast release (%)* | |
|---|---|---|---|
| Buffer | 72 | 2.02±0.36[a] | — |
| Intact W-256 cells | 24 | 2.81±0.51 | — |
| W-256 cell membranes | 24 | 10.03±0.79[b] | — |
| Intact W-256 cells | 48 | 8.91±0.30[b] | — |
| W-256 cell membranes | 48 | 24.21±0.36[b] | — |
| Intact W-256 cells | 72 | 17.57±1.88[b] | — |
| W-256 cell membranes | 72 | 65.99±6.49[b] | — |
| W-256 cell membranes+DFP | 72 | 44.66±4.53[b] | (−33.9±9.7[c]) |
| W-256 cell membranes+PMSF | 72 | 39.73±0.65[b] | (−41.3±6.2) |

[a]Mean±s.d.
[b]$P<0.001$ as compared to controls.
[c]See footnote to **Table I**.

Walker 256 cell membranes were prepared by hypotonic lysis followed by sucrose density gradient centrifugation and characterized by marker enzyme analysis and electron microscopy as previously described (DiStefano *et al.*, 1982). Walker 256 cell membranes (100 μg ml$^{-1}$) or intact W-256 cells were incubated with $^{125}$I-labelled L929 fibroblasts ($5 \times 10^6$ ml$^{-1}$) for 1, 2 or 3 days and fibroblast lysis was measured as noted in **Table I**. The 72 h experiment included a concurrent incubation of W-256 cell membranes with DFP ($10^{-3}$ M) or PMSF ($2 \times 10^{-4}$ M). These enzyme inhibitors alone did not affect the baseline release of $^{125}$I from fibroblasts.

**Table III** The effect of proteinase inhibitors on the invasiveness of Walker 256 cancer cells in urinary bladder organ cultures from rats

| *Pharmacologic agents* | *Concentration* (*M*) | *Invasion index* (%) | |
|---|---|---|---|
| None (control) | | $54.9 \pm 1.8^{a}$ | |
| DFP | $10^{-3}$ | $17.3 \pm 4.4^{b}$ | $(-68.4 \pm 8.0^{d})$ |
| TLCK | $5 \times 10^{-4}$ | $33.7 \pm 8.3^{b}$ | $(-38.6 \pm 15.2)$ |
| PMSF | $2 \times 10^{-4}$ | $37.9 \pm 7.9^{c}$ | $(-30.9 \pm 14.5)$ |
| Leupeptin | $10^{-3}$ | $55.3 \pm 11.7$ | $(+0.7 \pm 21.5)$ |
| Copper | $10^{-3}$ | $48.4 \pm 12.2$ | $(-11.8 \pm 22.4)$ |
| 1, 10 Phenanthroline | $10^{-3}$ | $69.1 \pm 8.4$ | $(+25.8 \pm 15.8)$ |
| Emetine | $10^{-4}$ | $42.1 \pm 2.9$ | $(-23.3 \pm 5.8)$ |
| EACA | $2 \times 10^{-3}$ | $53.7 \pm 0.4$ | $(-2.1 \pm 3.2)$ |

[a]Mean ± s.d.
[b]$P < 0.001$ as compared to controls.
[c]$P < 0.05$ as compared to controls.
[d]See footnote to **Table I.**

Isolated bladders from male Wistar rats were given injections of $10^6$ viable $^{125}$I-UdR labelled W-256 cancer cells that had been preincubated for 30 min with non-toxic concentrations of 7 different proteinase inhibitors and emetine. Organ cultures were incubated for 2 days at 37°C after which the bladders were removed from the tubes and radioactivity of the external fluid bathing the bladder (representing cells penetrating the full thickness of the organ) and the intact bladder were separately measured in a gamma counter. $^{125}$I radioactivity associated with the tumour cells which had penetrated through the bladder into the external fluid was expressed as a percentage of the initial total cellular radioactivity (Invasion Index). The results are compiled from 10 different experiments in which buffer treated W-256 cells were compared to 2–3 different proteinase-inhibitor treated tumour cells. The number of bladders tested per treatment group varied between 6 and 20.

membrane, fibrous connective tissue stroma and muscle components (Poste *et al.*, 1980). In the initial experiments we demonstrated that 40–60% of $^{125}$I-labelled W-256 cells penetrated the rat urinary bladder during a 48 h incubation. By contrast, 2% of alcohol-treated W-256 cells and 8% of $^{125}$I-UdR-labelled L929 fibroblasts (a non-invasive cell line) had penetrated the bladder in a 48 h period thus supporting the resistance of the urinary bladder to penetration by dead tumour cells or non malignant cells. Pretreatment of W-256 cancer cells with non-toxic concentrations of DFP, PMSF or TLCK inhibited bladder wall penetration by 68%, 31% and 39%, respectively (Table III). ε-amino caproic acid (EACA), $Cu^{++}$, leupeptin, (an arginine containing peptide analogue inhibitor of serine and cysteine proteinases, especially cathepsin B), and 1, 10 phenanthroline, did not affect W-256 invasion of the bladder. Short-term incubation with emetine inhibited bladder invasion by 23%.

To ascertain whether the passage of $^{125}$I through the urinary bladder represented intact cancer cells with proliferative capacity of $^{125}$I release from damaged cancer cells, we examined the colony forming ability in agar of W-256 cells that had penetrated through the urinary bladder. Two days after instilling $10^6$ cancer cells into the bladder, 406 + 31 W-256 colonies were noted in the surrounding semisolid media. Pretreatment of the W-256 cells with DFP resulted in 74 + 19 colonies per plate which indicated 82% inhibition of invasion. As noted above, DFP alone did not inhibit W-256 colony formation. Based on these pharmacologic experiments, we conclude that W-256 cancer cell serine proteinases are essential for cancer invasion through a urinary bladder.

Plasminogen-dependent fibrinolytic activity of W-256 cancer cells and the effect of various proteinase inhibitors on cancer cell PA was determined in an 18 h assay using $^{3}$H-fibrinogen and human plasminogen (Table IV). As anticipated, W-256 cell membranes and whole cell homogenates were rich in PA activity, which could be abrogated by pre-treatment with EACA, DFP, SBTI (soybean protein inhibitor of trypsin-like enzymes), and TLCK. Since EACA, a plasmin antagonist was able to completely inhibit fibrinolysis in this experiment, without affecting bladder invasion (Table III) or fibroblast target lysis (Table I), it appears that W-256 cancer invasion is unrelated to plasminogen activation. In contrast, the more general serine proteinase inhibitors (DFP, PMSF) were able to inhibit both the fibrinolytic and invasive aspects of W-256 cancer cells.

**Table IV** Plasminogen-dependent fibrinolytic activity of Walker 256 cancer cells and the drug-inhibitory profile

| *Sample* | *$^{3}$H-Fibrin release % per μg sample* | *% Inhibition of fibrinolysis* |
|---|---|---|
| Buffer | 0 | |
| Urokinase (1Ploug unit) | 100 | |
| W-256 homogenate | 38.8 | |
| W-256 cell membranes | 46.1 | |
| W-256 membrane + DFP | 9.1 | 80 |
| W-256 membrane + TLCK | 9.3 | 80 |
| W-256 membrane + EACA | 0 | 100 |
| W-256 membrane + SBTI | 0 | 100 |

$^{3}$H-fibrin was clotted on petri dishes using foetal calf serum with the addition of human plasminogen. Walker 256 cell homogenates and cell membranes (1 μg samples) were then added. After 18 h of incubation at 37°C the release of solubilized fibrinopeptides was measured. The release of $^{3}$H by tumour cell fractions was compared to maximum release produced by urokinase (1 ploug unit = 100%). The serine protease inhibitors, DFP, TLCK, EACA at concentrations shown in **Table I** and SBTI ($2 \times 10^{-5}$ M) were preincubated with W-256 cell membranes for 30 min prior to performing the standard fibrinolytic assay.

**Table V** Collagenase-like activity of W-256 cancer cells as measured by the hydrolysis of the synthetic substrate for collagenase DNP-Pro-Gln-Ile-Ala-Gly-Gln-D-Arg-OH (Peptide P)

| *Sample* | *Concentration (μg protein/assay)* | *Rate of hydrolysis ($OD_{365}$/18 h)* | *Specific activity (OD/mg)* |
|---|---|---|---|
| Solublization buffer | 0 | 0.007 ± 0.003 | — |
| W-256 whole cell homogenate | 58.8 | 0.249 ± 0.025 | 0.0042 |
| W-256 cell membranes | 60.5 | 0.275 ± 0.013 | 0.0045 |
| Bacterial collagenase | 400 (60 U) | 0.234 ± 0.009 | 0.00058 |
| Trypsin | 100 | 0 | 0 |

$5 \times 10^{-4}$ M DNP-Peptide P was dissolved in buffer and incubated with an equal volume of tumour cell homogenate, cell membrane (Triton X-100 solubilized), bacterial collagenase, or trypsin. After 18 h the enzyme reaction was stopped and the released DNP-Peptide fragments were determined by measuring the absorbance (OD 365) in the organic layer.

Collagenolytic activity of W-256 cells was assessed using two different approaches. Table V shows that W-256 cell homogenates and isolated cell membranes possess considerable collagenase-like activity, as monitored using the synthetic substrate, Peptide P, without enzyme enrichment in the cell membrane fraction as assessed by specific activity. To determine the category of enzyme(s) mediating this collagenase-like activity, we preincubated W-256 cell membranes with inhibitors of the metallo, serine and cysteine proteinases (Table VI). The metalloenzyme inhibitors 1,10 phenanthroline, EDTA and dithiothreitol effectively inhibited the hydrolysis of Peptide P (100%, 100% and 70% respectively). The serine proteinase (DFP, PMSF) and cysteine proteinase inhibitors (N-ethylmaleimide, leupeptin) had no significant effect of Peptide P hydrolysis. Since peptidases without collagenase activity can also cleave Peptide P (Gray & Saneli, 1982), a specific assay for collagenase was performed using soluble Type I $^{3}$H-collagen. Walker 256 cells were rich in latent collagenase activity producing 15.9% degradation of substrate mg$^{-1}$ protein with considerable enrichment (6.2×) in the plasma membrane fraction (99% degration mg$^{-1}$ protein). The metalloenzyme inhibitors, EDTA and 1,10 phenanthroline, produced >90% inhibition of W-256 cell collagenolytic activity.

**Table VI** Requirement for metalloenzyme activity in W-256 cell membrane induced hydrolysis of Peptide P

| *Sample* | *Pharmacologic agents* | *Rate of hydrolysis (0D/18 h)* | *(%)* |
|---|---|---|---|
| Walker 256 membrane | None | 0.126 | (—)[a] |
| Walker 256 membrane | DFP ($10^{-3}$ M) | 0.120 | (−5) |
| Walker 256 membrane | 1,10 phenanthroline ($10^{-2}$ M) | 0 | (−100) |
| Walker 256 membrane | N-ethyl maleimide ($10^{-3}$ M) | 0.123 | (−2) |
| Walker 256 membrane | PMSF ($2 \times 10^{-4}$ M) | 0.128 | (+2) |
| Walker 256 membrane | EDTA | 0 | (−100) |
| Walker 256 membrane | Leupeptin ($10^{-3}$ M) | 0.125 | (−1) |
| Walker 256 membrane | DTT ($10^{-3}$ M) | 0.039 | (−69) |

[a]See footnote to **Table I.**

The effect of different protease inhibitors on the rate of hydrolysis of Peptide P after an 18 h incubation with W-256 cell membranes (66 μg) was measured as described in **Table V.** Pharmacologic agents were preincubated with the tumour cell membranes for 10 min before adding the mixture to Peptide P.

**Table VII** Latent collagenase activity of W-256 cancer cell homogenate and plasma membranes

| *Sample* | *Pharmacologic agent* | *$^3$H-Collagen degradation (% substrate mg$^{-1}$ protein)* | *% Inhibition of collagenolysis* |
|---|---|---|---|
| W-256 whole cell homogenate (WCH) | None | 15.9 | — |
| W-256 whole cell homogenate (WCH) | EDTA | 0.5 | 97 |
| W-256 whole cell homogenate (WCH) | 1,10 phenanthroline | 8.3 | 48 |
| W-256 plasma membranes | None | 99.0 | — |
| W-256 plasma membranes | EDTA | 9.1 | 90 |
| W-256 whole cell membranes | 1,10 phenanthroline | 11.7 | 88 |

Tumour cell fractions were activated with trypsin-TPCK, inactivated with SBTI and then incubated at 27°C for 36 h with 10 μg ($^3$H-methyl) Type I collagen. Undigested collagen was then precipitated by the addition of dioxane and radioactivity in the filtrate containing collagen degradation products was measured. In the absence of trypsin activation, W-256 cell homogenate lacked measurable amounts of collagenase activity.

Minimal collagenase activity was noted in W-256 cell fractions without prior trypsin activation (Table VII).

### Discussion

The direct interaction between cancer cells and normal cells during the invasive process has been a difficult problem for experimental evaluation (Mareel, 1981). Taking advantage of the enhanced susceptibility of erythroblasts and red blood cells to experimental lysis, Zucker & Lysik (1977) introduced the tumour-induced erythroid cytolysis assay (TIEC) to assess the capacity of cancer cells to destroy normal host cells. All 13 cancer cell lines tested to date have been capable of lysing normal $^{59}$Fe-labelled bone marrow and red blood cells (Lysik *et al.*, 1979: DiStefano *et al.*, 1983*b*; Stevens *et al.*, 1983). Using highly invasive W-256 rat cancer cells, DiStefano *et al.* (1983*b*) were able to localize and identify serine proteinases in the cancer cell membrane that were responsible for the lysis of normal erythroid cells. One of these W-256 membrane enzymes, Memsin, has been purified and characterized as a highly active trypsin-like serine proteinase (LaBombardi *et al.*, 1983).

In this report we have shown that intact W-256 cancer cells are able to lyse $^{125}$I-UdR labelled fibroblasts by a cell contact requiring phenomenon. Target cell release of $^{125}$I into the culture media represents loss of DNA from the dying cell. Isolated cancer cell membranes were highly enriched in cytolytic function. Based on the inhibition of tumour-induced fibroblast lysis by non-toxic concentrations of DFP and PMSF, we conclude that W-256 cancer cell serine proteinases with trypsin-like specificity and localization in the cell membrane are responsible for the destruction of fibroblasts *in vitro*. The absence of fibroblast lysis when tumour cells were physically separated from $^{125}$I-labelled fibroblasts in culture and when fibroblasts were cultured in tumour-conditioned media serves to exclude the possibilities that nutritional deprivation or cancer cell secretion of toxic substances are causative factors in fibroblast lysis. Of interest, the inhibitory effect of 1,10 phenanthroline on tumour colony formation, DNA and protein synthesis and on fibroblast DNA and protein synthesis did not lead to diminished tumour-induced fibroblast lysis. This is consistent with our previous studies of W-256 induced erythroblast lysis, where we demonstrated that agents which primarily inhibit DNA synthesis had no significant effect on tumour-induced RBC lysis (DiStefano *et al*., 1979). By contrast, the cytolysis inhibitory effect of emetine is probably due to the profound inhibition of tumour protein synthesis, presumably the cytolytic proteinases. Finally, we cannot exclude the possibility that the inhibition of fibroblast lysis produced by copper may be due to a toxic effect on tumour cells. *A propos* of our work, Sakiyama *et al.* (1984) have shown that metastatic cancer cells can solubilize and phagocytose fixed cells by a plasminogen-dependent cell contact requiring phenonemon. Cancer cell attack of fixed cells was abrogated by inhibitors of trypsin-like proteinases, suggesting that the degredation of target cell proteins is a serine proteinase requiring phenomenon.

To evaluate invasiveness through tissue components of an intact organ, we examined the penetration by cancer cells of an isolated rat urinary bladder over a 2 day period. In the reports by Hart (1979) and Poste *et al.* (1980) using a mouse urinary bladder and variant melanoma cell lines, a 7 day period was required for tumour cell penetration of the bladder. By contrast, rat W-256 cancer cells were highly invasive of a rat urinary bladder in a 2 day period. Using different categories of proteinase inhibitors we showed that bladder invasion required non plasminogen dependent serine proteinases, which is consistent with our data in the fibroblast lysis assay. Explanations for the effects of proteinase inhibitors on cancer cells other than a direct action on invasive enzymes cannot be excluded. Pharmacologic inhibition of cancer cell motility or aspects of invasion other than tissue destruction need to be considered.

In these experiments we confirmed that W-256 cancer cells possess metalloenzymes with type I collagenolytic activity (Wolf & Wirl, 1982). However, based on the ineffectiveness of metalloenzyme inhibitors in altering urinary bladder invasion or normal fibroblast cytolysis, we conclude that metalloenzymes are not crucial in these assays. The role of collagenases in W-256 cancer invasion *in vivo* cannot be directly extrapolated from the current study, especially in organ sites rich in type IV basement membrane. The metalloenzyme, collagenase, has been well characterized in many cancer cell lines. Liotta *et al.* (1980, 1981) demonstrated a positive correlation between cancer cell collagenase with specificity for type IV collagen, a component of vessel wall basement membrane, and the invasive and metastatic capacity of various cancer cells. While we have not demonstrated a requirement for metalloproteinases in W-256 cancer cell-induced lysis of fibroblasts or erythroid cells, in other studies employing murine B16 melanoma cell lines, cancer-induced target cell lysis required metalloproteinases as evidenced by an inhibition of cytolysis elicited by metal chelators (DiStefano *et al*., 1983*b*).

Although plasminogen activator was identified in W-256 cancer cells and membranes using a $^{3}$H-fibrinogen substrate with added plasminogen, the generation of plasmin in the fibroblast cytolysis assay did not appear to be a critical factor as evidenced by a lack of requirement for exogenous plasminogen and the absence of an inhibitory effect of EACA, a plasmin inhibitor, in target cell destruction.

The importance of different categories of proteolytic enzymes in cancer invasion and metastasis has been repeatedly stressed. Bossman *et al.* (1973) initially reported a positive correlation between the metastatic ability of B16 melanoma variants and an increase in tumour cell trypsin and cathepsin-like serine proteinases. Numerous correlations between cancer cell metastatic characteristics and plasminogen activator have been reported (see review by Duffy & O'Grady, 1984). Using antibodies against plasminogen activator in a chick embryo experimental model of metastasis, Ossowski & Reich (1983) demonstrated that plasminogen activator was crucial for metastasis by selected cells.

High levels of lysosomal and cell membrane cysteine proteinases (Cathepsin B) have been found in cancer cell lines with enhanced metastatic capability (Recklies *et al*., 1982; Sloane *et al*., 1981; Pietra & Roberts, 1981). We have found that although W-256 cancer cells have cysteine proteinase activity (data not shown), the inhibition of this category of proteinases by leupeptin had no effect on the *in vitro* invasion assays that we employed here.

These studies reemphasize the extreme biological variability of cancer in that an individual cancer cell may contain several types of proteinases which may or may not correlate with metastatic potential. Recently Lowe & Isaacs (1984) reported that prostatic cancer cell lines of low and high metastatic potential possess a large array of proteinases with no single enzyme activity correlating with metastatic potential.

To date, the goal of most investigations has been to determine a correlation between a category of cancer proteins (hydrolytic enzymes, surface glycoproteins, etc.) and metastatic or invasive potential of cancer cell lines. In this study we have shown that pharmacologic agents can be used to better characterize the role of specific proteinases in experimental models that examine various factors involved in cancer invasion and normal cell destruction *in vitro*. The potential use of proteinase inhibitors in the treatment of cancer has not yet been systematically explored (Nelles & Schnebli, 1982). Our report suggests that the selection of enzyme inhibitors for future *in vivo* testing should be based on *in vitro* assays using the specific cancer cell line to be evaluated.

Supported by Merit-Review Research Funds from the Veterans Administration. The authors are grateful to Ms Janine M. Wieman and Dean P. Wilkie for their assistance in completion of this manuscript.

### References

BARRETT, A.J. (1980). The cellular proteinases – A broad view. In *Proteinases and Tumor Invasion*, p. 56. (Ed. Baici). Raven Press, New York.

BARRETT, J.C., CRAWFORD, B.D. & POP, T.S.'o. (1977). Quantitation of fibrinolytic activity of Syrian hamster fibroblasts using $^3$H-labeled fibrinogen prepared by reductive alkylation. *Cancer Res.*, **37**, 1182.

BOSSMAN, H.B., BIEBER, G.F., BROWN, A.E. & HALL, T. (1973). Chemical parameters correlated with tumor cell implantation. *Nature*, **246**, 487.

BHATNAGAR, R. & DECKER, K. (1981). A collagenese assay using ($^3$H-methyl) collagen. *Biochem. Biophys. Meth.*, **5**, 147.

DISTEFANO, J.F., BECK, G., LANE, B. & ZUCKER, S. (1982). Role of tumor cell membrane-bound serine proteases in tumor-induced target cytolysis. *Cancer Res.*, **42**, 207.

DISTEFANO, J.F., BECK, G. & ZUCKER, S. (1983*a*). Characterization of tumor cell membrane serine proteases by polyacrylamide gel electrophoresis. *J. Histochem. Cytochem.*, **31**, 1233.

DISTEFANO, J.F., BECK, G., MEHLING, G. & ZUCKER, S. (1983*b*). Cancer invasion: Membrane proteases in human and B16 cancer induced normal cell destruction. *Clin. Res.*, **31**, 405A.

DISTEFANO, J.F., LYSIK, R. & ZUCKER, S. (1979). Pharmacologic studies of the mechanism of tumor-induced marrow cytolysis. *Cancer Res.*, **39**, 1193.

DUFFY, J.J., & O'GRADY, P. (1984). Plasminogen activator. *Eur. J. Cancer Clin. Oncol.*, **20**, 577.

GRAY, R.D. & SANELI, H.H. (1982). Characterization of vertebrate collagenase activity by high-performance liquid chromatography using a synthetic substrate. *Anal. Biochem.*, **120**, 339.

GRANT, G.A. & EISEN, A.Z. (1980). Substrate specificity of the collagenolytic serine protease from Uca pugulator: Studies with non collagenous substrates. *Biochemistry*, **19**, 6089.

HART, I.R., (1979). The selection and characterization of a invasive variant of the B16 melanoma. *Am. J. Pathol.*, **97**, 587.

KOBAYASHI, S. & NAGAI, Y. (1978). Human leucocyte neutral proteases, with special reference to collagen metabolism. *J. Biochem.*, **84**, 559.

LABOMBARDI, V.J., SHAW, E., DISTEFANO, J.F., BECK, G., BROWN, F. & ZUCKER, S. (1983). Isolation and characterization of a trypsin-like serine proteinase from the membranes of Walker 256 carcinosarcoma cells. *Biochem. J.*, **211**, 695.

LIOTTA, L.A., TRYGGVASON, K., GARBISA, S., GEHRON, P., ROBEY, G. & SHIGETO, A. (1981). Partial purification and characterization of a neutral protease which cleaves type IV collagen. *Biochemistry*, **20**, 100.

LIOTTA, L.A., TRYGGVASON, K., GARBISHA, S., HART, I., FOLTZ, C.M. & SHAFIE, S. (1980). Metastatic potential correlates with enzymatic degradation of basement membrane collagen. *Nature*, **284**, 67.

LOWE, F.C. & ISSACS, J.T. (1984). Biochemical methods for predicting metastatic ability of prostate cancer utilizing the Dunning R-3327 rat prostatic adenocarcinoma system as a model. *Cancer Res.*, **44**, 744.

LYSIK, R.M., CORNETTA, K., DISTEFANO, J.F. & ZUCKER, S., (1979). Bone marrow cytolysis induced by hepatoma, teratocarcinoma, and transformed fibroblasts. *Cancer Res.*, **39**, 30.

MAREEL, M.M. (1981). Recent aspects of tumor invasiveness. *Int. Rev. Exp. Pathol.*, **22**, 65.

MARKWELL, M.A.K. & FOX, L.F. (1978). Surface-specific iodination of membrane proteins of viruses and eucaryotic cells using 1,3,4,6-tetrachloro-3-alpha, 6-alpha-diphenylglycoril. *Biochemistry*, **17**, 4807.

MASUI, Y., TAKEMOTO, T., SAKA KIBARA, S., HORI, H. & NAGI, Y. (1977). Synthetic substrates for vertebral collagenase. *Biochem. Med.*, **17**, 215.

NELLES, L.P. & SCHNEBLI, H.P. (1982). Are proteinase inhibitors potentially useful in tumor therapy?, *Invasion Metast.*, **2**, 113.

NICHOLSON, G.L. (1979). Cancer metastasis. *Sci. Am.*, **240**, 66.

OSSOWSKI, L. & REICH, E. (1983). Antibodies to plasminogen activator inhibit human tumor metastasis. *Cell*, **35**, 611.

PIETRAS, R.J. & ROBERTS, J.A. (1981). Cathepsin B-like enzymes – Subcellular distribution and properties in neoplastic and control cells from human ectocervix. *J. Biol. Chem.*, **256**, 8536.

POSTE, G. & FIDLER, I.J. (1980). The pathogenesis of cancer metastasis. *Nature*, **283**, 139.

POSTE, G., DOLL, J., HART, I.R. & FIDLER, I.J. (1980). *In vitro* selection of murine B16 melanoma variants with enhanced tissue-invasive properties. *Cancer Res.*, **40**, 1636.

QUIGLEY, J.P. (1979). Phorbol ester-induced morphological changes in transformed chick fibroblasts: Evidence for direct catalytic involvement of plasminogen activator. *Cell*, **17**, 131.

RECKLIES, A.D., MORT, J.S. & POOLE, A.R. (1982). Secretion of a thiol proteinase from mouse mammary carcinomas and its characterization. *Cancer Res.*, **42**, 1026.

SAKIYAMA, H., NISHINO, Y., NISHIMURA, K., NODA, Y. & OTSU, H. (1984). Phagocytosis and solubilization of fixed cells by hamster embryo fibroblasts, Nil 2C2. *Cancer Res.*, **44**, 2023.

SALO, T., LIOTTA, L.A. & TRYGGVASON, K. (1983). Purification and characterization of a murine basement membrane collagen-degrading enzyme secreted by metastatic tumor cells. *J. Biol. Chem.*, **258**, 3058.

SLOANE, B.F., DUNN, J.R. & HONN, K.V. (1981). Lysosomal cathepsin B correlation with metastatic potential. *Science*, **212**, 1151.

STEVENS, T.S., HULLEY, T.P., GRIFFIN, M.M. & ITZHAKI, S. (1983). Evidence for metal inhibition of tumor membrane-bound neutral protease and the control of tumor-induced target cell cytolysis. *Br. J. Cancer*, **46**, 850.

STRAYLI & WEISS, L. (1977). Cell locomotion and tumour penetration. *Eur. J. Cancer*, **13**, 1.

WOLF, L. & WIRL, G. (1982). Collagenase in the Walker carcinoma. A study of latent and active enzyme *in vivo* and *in vitro*. *Eur. J. Biochem.*, **121**, 623.

ZUCKER, S. & LYSIK, R.M. (1977). Cancer-induced cytolysis of normal bone marrow cells. *Nature*, **265**, 736.

ZUCKER, S., LYSIK, R.M. & DISTEFANO, J.F. (1980). Cancer cell inhibition of erythropoiesis. *J. Lab. Clin. Med.*, **96**, 770.